# Oscillatory markers of neuroHIV-related cognitive impairment and Alzheimer’s disease during attentional interference processing

**DOI:** 10.18632/aging.204496

**Published:** 2023-01-19

**Authors:** Chloe E. Meehan, Mikki Schantell, Alex I. Wiesman, Sara L. Wolfson, Jennifer O’Neill, Sara H. Bares, Craig M. Johnson, Pamela E. May, Daniel L. Murman, Tony W. Wilson

**Affiliations:** 1 Institute for Human Neuroscience, Boys Town National Research Hospital, Boys Town, NE 68010, USA; 2 Department of Psychology, University of Nebraska – Omaha, Omaha, NE 68182, USA; 3 College of Medicine, University of Nebraska Medical Center, Omaha, NE 68198, USA; 4 Montreal Neurological Institute, McGill University, Montreal, QC H3A 2B4, CA; 5 Geriatrics Medicine Clinic, UNMC, Omaha, NE 68198, USA; 6 Department of Internal Medicine, Division of Infectious Diseases, UNMC, Omaha, NE 68198, USA; 7 Department of Radiology, UNMC, Omaha, NE 68198, USA; 8 Department of Neurological Sciences, UNMC, Omaha, NE 68198, USA; 9 Memory Disorders and Behavioral Neurology Program, UNMC, Omaha, NE 68198, USA; 10 Department of Pharmacology and Neuroscience, Creighton University, Omaha, NE 68178, USA

**Keywords:** neuroHIV, magnetoencephalography, MEG, oscillations, top-down

## Abstract

People with HIV (PWH) frequently experience mild cognitive decline, which is typically attributed to HIV-associated neurocognitive disorder (HAND). However, such declines could also be a sign of early Alzheimer’s disease (AD) in older PWH. Distinguishing these two pathologies in PWH is exceedingly difficult, as there is a major knowledge gap regarding their neural and neuropsychological bases. In the current study, we begin to address this knowledge gap by recording magnetoencephalography (MEG) during a flanker interference task in 31 biomarker-confirmed patients on the AD spectrum (ADS), 25 older participants with HAND, and 31 cognitively-normal controls. MEG data was examined in the time-frequency domain using a data-driven approach. Our results indicated that the clinical groups (ADS/HAND) performed significantly worse than controls on the task and exhibited aberrations in interference-related theta and alpha oscillations, some of which were disease-specific. Specifically, patients (ADS/HAND) exhibited weaker interference activity in frontoparietal and cingulate cortices compared to controls, while the ADS group exhibited stronger theta interference than those with HAND in frontoparietal, occipital, and temporal cortices. These results reveal overlapping and distinct patterns of neurophysiological alterations among those with ADS and HAND in attentional processing centers and suggest the existence of unique oscillatory markers of each condition.

## INTRODUCTION

People with HIV (PWH) now have a normal life expectancy due to combination antiretroviral therapy (cART) and other modern medical advances. Despite the normal life expectancy, PWH remain at an elevated risk for developing various comorbidities, including the emergence of cognitive impairment as seen in HIV-associated neurocognitive disorder (HAND). HAND affects approximately 50% of PWH, irrespective of age [1, 2]. When such cognitive impairments emerge early (e.g., 3^rd^ or 4^th^ decade of life), the likelihood that they are related to HIV infection is generally high because the prevalence of other disorders leading to cognitive impairment is generally low in this age range. Conversely, neurological conditions such as Alzheimer’s disease (AD) commonly emerge later in life [3], and thus, the underlying cause of the emergence or worsening of cognitive impairment in older PWH is far less clear.

Whether related to HAND, AD spectrum (ADS) pathologies, or another neurological condition, identifying the origin of cognitive impairment in older PWH is challenging considering the substantial overlap in neurocognitive deficits, including deficits in the domains of working memory, attention, and visuospatial processing [3–5]. Combining cognitive testing and amyloid imaging using positron-emission tomography (PET) has become the gold-standard diagnostic approach for AD. However, in PWH, imaging studies of beta-amyloid have revealed normal deposition, irrespective of cognitive status, comparable to that seen in healthy demographically-matched adults [6–8]. Such differences in beta-amyloid deposition suggest AD and HAND have at least partially distinct pathological features, and amyloid imaging may be a reliable means to distinguish the conditions, albeit invasive and cost-prohibitive.

Apart from amyloid PET, many structural neuroimaging studies have shown decreases in cortical volume and thickness in HAND and ADS populations, particularly within the posterior cortices [9, 10]. Functional MRI (fMRI) studies have also reported reduced brain activation across frontoparietal regions during attentional processing in participants on the ADS relative to healthy controls [11–13], while similar studies in those with HAND have identified aberrant increases in activation within the prefrontal, parietal, and occipital cortices [14, 15]. Thus, structural and functional MRI studies have provided potential evidence that neural abnormalities in brain structure and function may both overlap and differ in ADS and HAND pathologies, but studies directly comparing the two groups are currently very rare.

Similar to the existing structural and functional MRI literature, there are ample studies using magnetoencephalography (MEG) in HAND and ADS groups individually, but few directly comparing the two conditions. MEG enables quantification of multi-spectral oscillatory activity including responses in the theta (4-8 Hz) and alpha (8-14 Hz) bands, which are known to critically support attention processing [16–19]. Further, recent work has shown that MEG responses are stable over at least three years in adults [20, 21] and vary systematically with mental/task states [22], both of which are critical for the development of neural markers of disease. However, less is understood about the impact of disease progression on MEG responses. Many of the MEG studies examining patients on the ADS and those with HAND have focused on resting-state activity [23–28], and far fewer MEG studies have used task-based paradigms to examine disease-specific neural aberrations supporting cognitive processes. The limited previous MEG studies of selective attention in the context of HIV have found altered theta and alpha activity, with theta predicting neuropsychological outcomes and distinguishing cognitively impaired and unimpaired PWH [29]. Though no task-based MEG studies of selective attention in ADS exist, recent EEG studies have found alpha activity to be altered in ADS disorders during attentional processing and recent resting-state MEG studies in AD have found consistent links between low-frequency activity and attention and processing speed [27, 28, 30, 31]. Given the limited work in this area, it is not surprising that no studies to date have directly examined the potentially shared and disease-specific aberrations in neural circuitry serving attention in those with HAND versus ADS. AD is not often diagnosed in older PWH reporting cognitive decline, as the emergence of such impairments are typically assumed to be HAND, despite reports of AD in older PWH with cognitive dysfunction [32, 33]. Therefore, finding noninvasive markers capable of dissociating cognitive dysfunction due to HIV or ADS is of utmost importance and would help launch the field toward new therapeutic approaches for the pathologically-specific features of each disorder.

In this study, we recorded task-based MEG to examine the oscillatory dynamics underlying visual selective attention in participants with HAND and those on the ADS*.* Attentional deficits are well-documented in those with HAND [15, 34], and engage similar regions across the frontoparietal network typically noted as having high amyloid deposition in patients on the ADS [35]. Given these deficits, all participants completed an arrow-based Eriksen flanker [36] selective attention task during MEG recording. Based on previous literature independently investigating the neural processes supporting visual attention in HAND and ADS pathologies [15, 29, 31, 37], we predicted that our primary findings would center on parieto-occipital and prefrontal regions, which are strongly activated during selective attention processing. More specifically, we hypothesized that those on the ADS would exhibit stronger neural interference responses than those in the HAND group, and that such differences would occur in the theta range within frontoparietal brain regions.

## RESULTS

### Behavioral analysis

**Figure 1:**
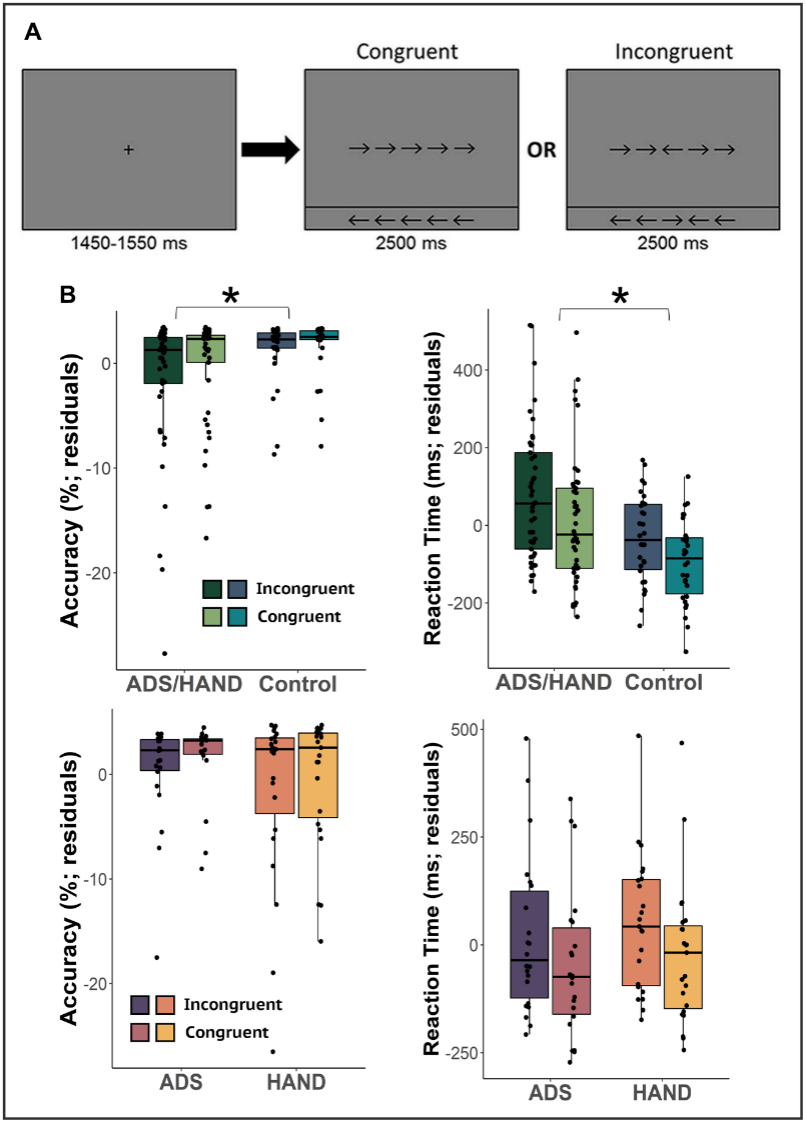
**Flanker attention task and behavioral metrics.** (**A**) A fixation cross was presented for 1,500 (± 50) ms followed by a row of 5 arrows for 2,500 ms. Participants were instructed to indicate whether the middle arrow was pointing to the left (right index finger) or right (right middle finger) via button press. (**B**) Residuals of reaction time and accuracy accounting for the effect of age for each condition are given on the y-axis and group on the x-axis. Cognitively impaired participants (ADS/HAND groups) were less accurate and slower to respond than cognitively normal adults. However, those on the ADS generally performed similar to participants with HAND. *p<.05.

Eighty-seven total participants were enrolled and matched group-wise on key demographic variables with the exception of age. Thus, age was included as a nuisance covariate in all statistical modeling. Participants performing poorly on the task (i.e., accuracy < 60%) were excluded from all MEG analyses. This resulted in the exclusion of nine participants in the ADS group and two in the HAND group. In addition, one control participant was also removed due to low quality MEG data (i.e., large magnetic artifacts). Thus, the final sample included 22 participants on the ADS, 23 with HAND, and 30 healthy controls. Full demographic information is provided in Table 1. First, we collapsed across both clinical groups (ADS + HAND) to examine behavioral performance in those with cognitive impairment relative to healthy controls. Our results indicated that patients were less accurate (F_1,72_ = 9.53, *p* = .003) and responded slower (F_1,72_ = 4.44, *p* = .039) than controls (Figure 1). Next, we compared the ADS and HAND groups and found no differences in reaction time (*p* = .442, *BF_01_* = 1.60) nor accuracy (*p* = .106 *BF_01_* = 0.90; Figure 1).

**Table 1:** Demographic Information.

	**ADS (n=22)**	**HAND (n=23)**	**Controls (n=30)**
Age (years)	69.41 (4.84)	58.48 (6.05)	65.60 (7.28)
Sex (% Female)	50%	53.33%	52.17%
Education (years)	14.68 (2.95)	13.13 (2.01)	16.73 (2.63)
ADL (% declined)	100%	69.57%	0%

ADS, Alzheimer’s disease spectrum; HAND, HIV-associated neurocognitive disorder; controls, cognitively normal controls; ADL, Activities of daily living.

### MEG sensor-level analyses

**Figure 2:**
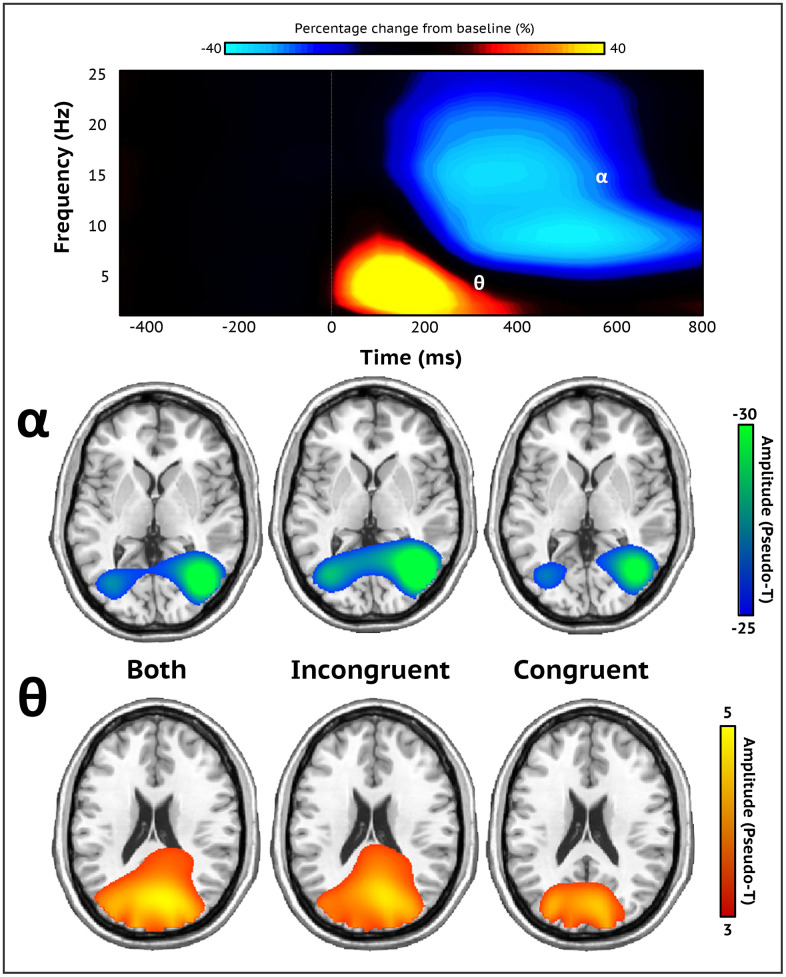
**Sensor-level spectrograms and whole-brain averages.** A time-frequency spectrogram illustrating task-specific oscillatory activity averaged across all trials, conditions, and participants (top). Time (ms) is on the x-axis and frequency (Hz) is presented on the y-axis. The color bar above the spectrogram indicates the percent change in amplitude from baseline. Robust increases in theta (3-6 Hz; 0-350 ms) and decreases in alpha activity (8-16 Hz; 200-600 ms) were found in occipito-parietal sensors (MEG1932). Both oscillatory responses significantly differed from baseline (*p* < .001, corrected). Stimulus onset (0 ms) is indicated by the dotted green line. Each brain image depicts the grand average across all participants of both, incongruent, and congruent trial conditions for each neural response (bottom). Strong theta oscillations were generated by neuronal populations in the bilateral primary visual cortices, while alpha oscillations were distributed across the lateral occipital cortices bilaterally. The color scale bar per neural response appears to the right in amplitude per voxel.

Similar to previous MEG studies utilizing the flanker task [29, 38], we identified two significant clusters of neural oscillatory activity via the sensor-level spectrograms (Figure 2). Specifically, we observed early increases in theta activity (3-6 Hz; 0-350 ms) followed by later, robust decreases in the alpha band (8–16 Hz) extending from 200 to 600 ms. Both oscillatory responses were strongest in posterior sensors near the parietal and occipital cortices (both *p’*s < .001, corrected). Note that the alpha response extended slightly beyond 600 ms, but given the earliest average reaction time we limited the imaging window to this latency to avoid capturing the motor response elicited by the button press.

### MEG source-level analyses

To identify the cortical regions generating these oscillatory responses, each significant time-frequency bin was imaged with a frequency-resolved beamformer for both conditions combined (e.g., incongruent and congruent) and each condition independently. The subsequent images were averaged across all participants per time-frequency response for incongruent and congruent conditions separately, as well as for both conditions together (Figure 2). These images indicated that the robust increases in theta activity were generated by populations of neurons in the bilateral visual cortices, while the strong decreases in alpha were distributed across lateral occipital regions bilaterally and right superior parietal cortices.

**Figure 3:**
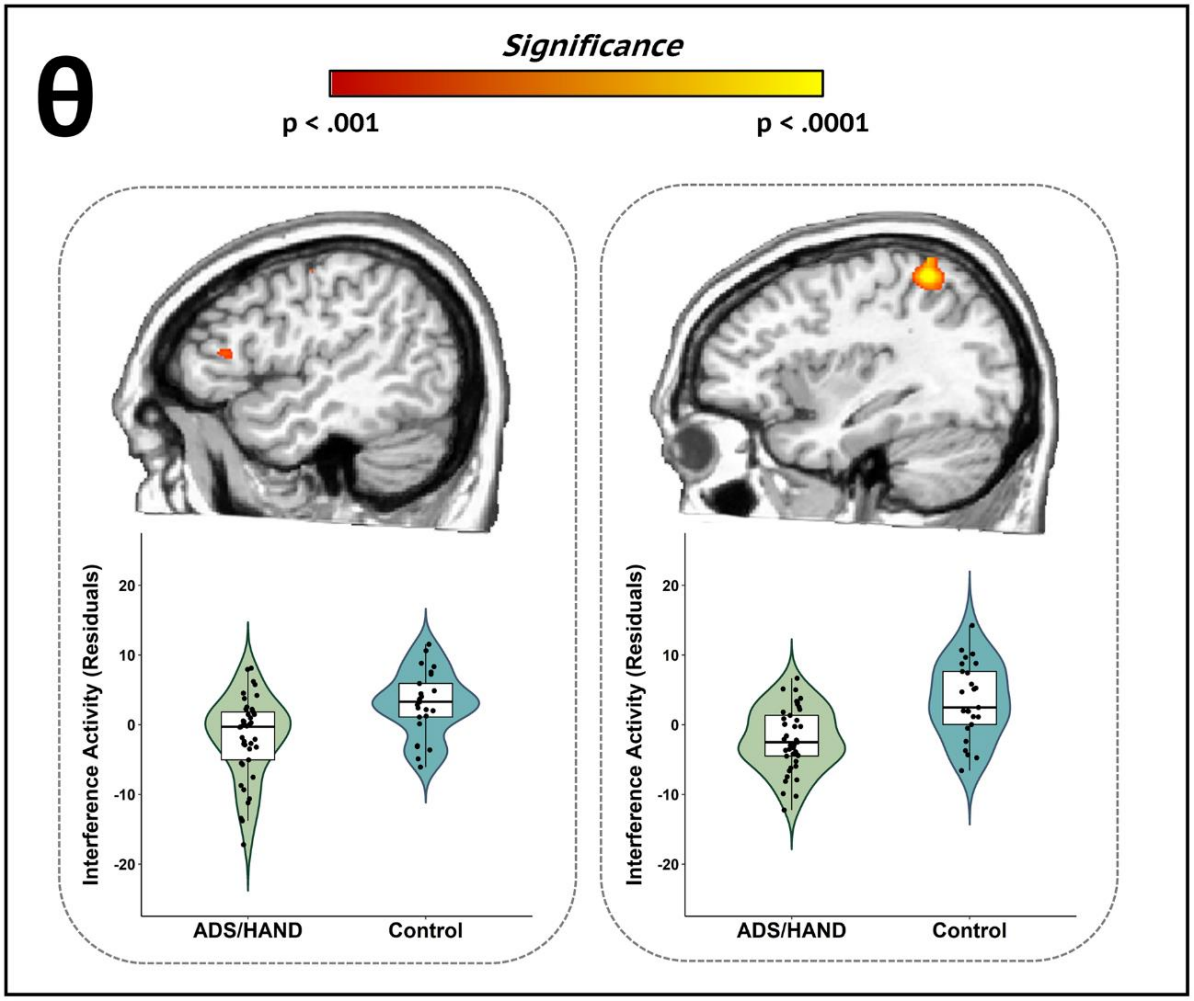
**Participants with ADS or HAND exhibited aberrant theta interference activity during attentional processing compared to healthy controls.** Whole-brain group difference maps of theta interference activity are displayed accompanied by violin plots for the peak voxel in each significant cluster. Group differences (p < .001, corrected) were revealed in left inferior frontal (left) and left superior parietal cortices (right). Residuals of amplitude values controlling for age are presented below each corresponding brain slice.

Given our hypotheses, we computed flanker interference maps (incongruent – congruent) per time-frequency response and subjected these maps to ANCOVAs with age as a nuisance covariate to identify oscillatory differences between patients (ADS + HAND) and controls. Patients exhibited weaker theta interference effects compared to controls in the left inferior frontal gyrus and left superior parietal cortex (Figure 3; all *p*s < .001, corrected). Post-hoc testing revealed that both ADS and HAND groups exhibited a weaker interference effect than controls in the left superior parietal cortex (all *p*s < .001), and that the ADS and HAND groups did not differ from each other (BF_01_ = 3.24). In the left inferior frontal cortex, the HAND group had a stronger theta interference effect than controls (*p* = .03), while participants on the ADS exhibited similar theta interference responses as both controls (BF_01_ = 2.46) and HAND (BF_01_ = .24) participants. In regard to alpha oscillations, controls exhibited stronger alpha interference effects in the right anterior cingulate cortex compared to patients (*p* < .001, corrected). Post-hoc testing indicated that both the ADS (*p* = .006) and HAND (*p* = .043) groups individually differed from controls but did not differ from each other in alpha interference activity (BF_01_ = 3.05).

**Figure 4:**
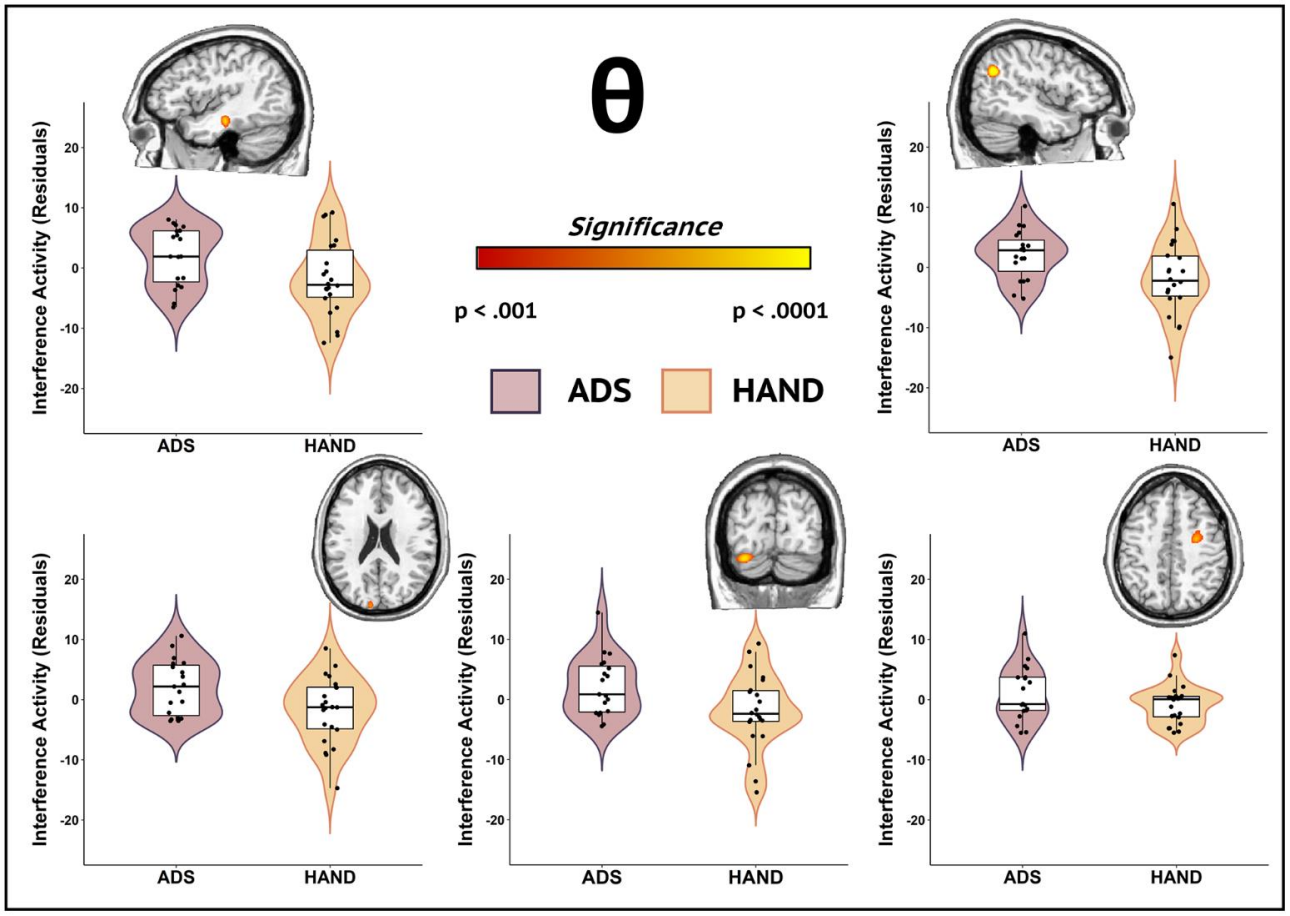
**Participants in the ADS group exhibited stronger theta interference activity compared to those in the HAND group during attentional processing.** Whole-brain group comparison maps of theta interference activity are shown. Images for each significant cluster of activity are accompanied by the violin plots of the amplitude values for the peak voxel. Group differences (*p* < .001, corrected) were identified in the left middle temporal gyrus (top left), right superior parietal cortex (top right), left middle occipital cortex (bottom left), right lingual gyrus (bottom middle), and right dorsolateral prefrontal cortex (bottom right). Residuals of amplitude values controlling for age are given with each corresponding brain slice.

To identify disease specific oscillatory aberrations (i.e., ADS vs. HAND), we utilized a whole-brain exploratory approach on the theta and alpha interference maps. Our key findings indicated that those in the ADS group exhibited stronger theta interference effects compared to participants in the HAND group (*p* < .001, corrected; Figure 4) across multiple cortical regions, including the left middle temporal gyrus, right superior parietal cortex, left middle occipital area, left lingual gyrus, and right dorsolateral prefrontal cortex. Alpha interference activity did not statistically differ by group in any region.

## DISCUSSION

There is currently an immense knowledge gap regarding the neural dysfunction and cognitive symptoms common among and differentiating between ADS and HAND pathologies. In this study, we utilized MEG to dissect the neural dynamics engaged during attentional processing in patients with HAND, those on the ADS spectrum, and cognitively normal controls. Our results indicated that both ADS and HAND groups exhibited region-specific differences relative to controls in theta and alpha interference responses across regions serving attention processing (e.g., frontoparietal and anterior cingulate cortices). Moreover, our exploratory analyses revealed disease-specific differences in theta interference activity across multiple cortical regions, such that those on the ADS exhibited stronger interference activity than the HAND group. The implications of these findings are considered below in detail.

Given that previous MEG studies of selective attention using the Flanker [36] task in healthy adults have highlighted the importance of theta and alpha neural oscillations [38], we fully anticipated the robust theta and alpha responses that were observed across the entire sample. One prior MEG study reported aberrant theta and stronger baseline alpha activity in the prefrontal cortices of participants with HAND, which distinguished cognitively impaired from unimpaired PWH and controls [29]. Other studies of attention processing in patients on the AD spectrum revealed decreased theta [39, 40] and alpha [30, 31] responses during attention and visuospatial processing. Thus, while the current findings are novel, the overlap in aberrant attention-related oscillatory activity in each frequency band across frontoparietal hubs in the ADS and HAND groups relative to controls is not entirely surprising. Briefly, the cognitively impaired groups (ADS + HAND) exhibited weaker theta interference effects compared to controls in the parietal cortex, along with stronger interference activity in the frontal cortex. Both of these neural regions are critical to attention function and these group differences may reflect both early and downstream effects, whereby aberrations early in the dorsal attention processing stream (e.g., parietal cortex) are offset by compensatory neural responses in frontal cortices to allocate additional attentional resources to maintain adequate task performance. Increases in theta activity have been linked to both the initial organization [16, 19] and hierarchical processing of attention [19, 41, 42], and thus theta interference responses during attentional processing may be an indicator of both primary and higher order processes, which in the current study were potentially both aberrant (i.e., early parietal activity) and compensatory (i.e., late frontal activity) across both HAND and ADS disease pathologies. Moreover, decreased alpha interference responses in the cognitively impaired groups (ADS + HAND) relative to controls were observed in the anterior cingulate cortex. Alpha activity has been linked to the allocation of attentional resources and the anterior cingulate cortices are known to be critical for top-down cognitive control [18, 19, 43]. Thus, such altered alpha interference responses during selective attention may suggest common dysfunction across both HAND and ADS in the higher-order subprocesses of attention allocation. The degree to which these alpha aberrations were offset by compensatory processing in the theta spectral range is unknown and should be a focus of future work.

Beyond the general differences between patients and controls, we found disease-specific theta alterations in several brain regions including the prefrontal, parietal, occipital, and temporal cortices. Theta aberrations have been highlighted in previous studies as markers of cognitive impairment separately in AD and HAND [27, 29, 40, 44, 45]. These disease-specific differences in theta oscillatory activity may suggest more fine-scale neurophysiological differences in temporal segmentation and higher order allocation of attentional resources, which could be secondary to regional amyloid accumulation in those on the ADS relative to HAND, as multiple studies have shown normal amyloid levels in patients with HAND [6, 8]. Further, aberrations in theta activity have also been linked to tau accumulation [46] in ADS cases, which is known to follow major amyloid accumulation. Although the cause of neural and cognitive deficits in HAND is uncertain, persistent neuroinflammation, mitochondrial dysfunction, and accelerated aging are all thought to play a major role [47–53], which separately or together may lead to altered theta oscillatory dynamics in attention circuitry. Regardless, our results suggest that theta oscillations are differentially affected across a network of brain regions in ADS compared to HAND, with the latter group exhibiting sharply decreased theta neural interference effects.

As briefly noted above, these disease-specific theta abnormalities were distributed across key brain areas serving attentional allocation and processing. Potentially the most interesting regions exhibiting disease-specific theta deficits were the right superior parietal and dorsolateral prefrontal cortices. Such right frontoparietal cortices are well-known for their role in the allocation and coordination of attentional resources [54–56], with parietal cortices thought to aid in attentional shifting [57] and prefrontal regions directing the higher order control of attention [58]. Disease-specific theta aberrations were also observed in the left middle occipital cortex and lingual gyrus, which are thought to direct and amplify visuospatial attention [59]. Considering the specific functions of these regions during attention processing, the presence of disease-specific deficits in theta oscillatory activity within these circuits is not entirely surprising [29, 30, 39, 40, 44]. In addition to the frontoparietal and visual cortices, we found neural responses in temporal regions distinguishing ADS and HAND. Theta oscillations occurring in temporal regions have previously been associated with top-down cognitive control and binding [60, 61]. Aberrant occipitotemporal structure and function have been attributed to visual attention deficits in AD [37, 62] and PWH [63, 64]. Thus, differences in the occipitotemporal regions may suggest deficient early binding of relevant stimulus information and may, in turn, create complications in the later higher order control of allocating attentional resources such as in disease-specific aberrations detected in the frontoparietal regions. However, the neurobiological origins of these deficits may differ between those with HAND and ADS, as only ADS pathology has been linked to amyloid deposition whereas as HAND has been attributed to a family of interconnected causes (e.g., inflammation, mitochondrial redox environment, etc.) [6, 8, 47–53].

Before closing, it is important to articulate the limitations of the current study. Foremost, only the ADS group consisted of participants who all underwent amyloid PET imaging. Although studies to date have broadly shown amyloid PET negativity in HAND [6–8], future studies should include amyloid PET for the entire sample, which would enable new avenues for the multimodal integration of PET and MEG when directly comparing ADS and HAND neuropathology. In addition, our sample size was only moderate and while being adequately powered for comparisons involving our three core groups (i.e., ADS, HAND, and cognitively-normal controls), we were underpowered to evaluate sex differences in the cognitive and neurophysiological parameters of interest. Examining sex differences in ADS and HAND is critical to advancing the field and should be a focus of future studies. Along the same lines, we were underpowered to probe differences among HAND subtypes (e.g., HIV-associated dementia versus mild neurocognitive disorder) and this should be a focus of future work in this area. Another limitation is that we did not consider other biological metrics that may be disease-specific, such as blood-based indicators of neuroinflammation and/or the mitochondrial redox environment. Although the cause of HAND remains uncertain, neuroinflammation is a leading concern [47, 65] and neuroimmunological factors are also thought to contribute to ADS pathogenesis [66, 67]. Thus, future studies should integrate inflammatory marker panels and other blood-based assessments to identify disease-specific biological, neurophysiological, and interactive processes that lead to cognitive decline. Additionally, impairment in AD and HAND extends across numerous cognitive domains beyond attention, including motor control and working memory [3, 15, 68, 69]. Thus, investigating the neural dynamics underlying other cognitive domains may be of particular interest as a future direction. Alpha and theta activity are also known to support working memory, motor processing, and emotion regulation [70–74], which are cognitive functions known to be aberrant in both AD and HAND independently [27, 29, 40, 44].

In conclusion, the current study examined the commonalities and differences in neurophysiological processing underlying cognitive dysfunction in persons with HAND and those on the ADS. Our primary findings included theta and alpha interference effects in frontoparietal regions that differentiated cognitively-impaired participants (i.e., those with ADS and HAND) from cognitively normal controls. In addition, we identified disease-specific theta interference responses in key attention processing areas that distinguished patients on the ADS from those with HAND. In sum, the current study is the first to probe the commonalities and differences in the neural dynamics serving attention in ADS and HAND, and our most important findings suggest that some neural oscillatory deficits are common across the two conditions, while others are likely specific to the neuropathology of each disorder.

## MATERIALS AND METHODS

### Participants

Thirty-one amyloid-positive patients on the Alzheimer’s disease spectrum with amnestic mild cognitive impairment (aMCI) or mild probable AD, as determined by a fellowship-trained neurologist specializing in memory disorders, were enrolled in this study. Twenty-five cognitively-impaired PWH who were receiving effective cART and were virally suppressed at the time of the study also participated in this study. In addition, a control group of 31 older adults with normal cognition were enrolled to serve as reference sample for the standard, task-dependent neural dynamics implicated in healthy aging. All participants were between the ages of 51 and 79 years at the time of enrollment and were recruited through two different projects (R01-MH116782-S1 and R01-MH118013-S1). The groups were matched on key demographic variables with the exception of age, as the HAND group was slightly younger than healthy controls and ADS patients. Note that age was included as a nuisance covariate in all statistical modeling. Exclusion criteria included any medical illness affecting CNS function, any neurological disorder (other than AD/aMCI/HAND), history of head trauma, and current substance use disorder. Written informed consent was obtained from each participant and their informant (if applicable) following a detailed description of the study. In cases where capacity to consent was questionable, educated assent was obtained from the research participant, in addition to informed consent from a legally-authorized representative.

### Neuropsychological testing

All participants underwent a battery of neuropsychological assessments (Table 2), with raw scores for each participant being converted to demographically-adjusted z-scores using published normative data [75–77]. This battery, which was developed in collaboration with a clinical neuropsychologist specializing in cognitive disorders, assessed multiple functional domains known to be impaired in patients with HAND and those on the AD spectrum. Specifically, the cohort of PWH were assessed on the following cognitive domains per the Frascati criteria [68] learning, memory, attention and executive function, motor, and processing speed. The ADS cohort completed a neuropsychological assessment that assessed commonly impaired cognitive domains in AD: learning, memory, attention and executive function, language, and processing speed. In addition, we measured premorbid function and functional impairment in all participants, along with general cognitive status in the AD group. Controls completed one of these two batteries depending on which project they were drawn from. Using these assessments and activities of daily living (ADL) [78], PWH were diagnosed with HAND according to the Frascati guidelines, including subgroups with asymptomatic neurocognitive impairment (ANI; 16 patients or 69.57% of final sample; i.e., having at least two cognitive domains one SD below the standardized mean with no functional declines), mild neurocognitive disorder (MND; two patients or 8.70% of final sample; i.e., at least two cognitive domains one SD below the standardized mean, with ADL deficits), or HIV-associated dementia (HAD; five patients or 21.74% of final sample; i.e., having at least two cognitive domains two SDs below the standardized mean, with ADL deficits). Healthy controls were cognitively normal and did not meet the above criteria for neuropsychological impairment. For patients on the ADS, instrumental activities of daily living (IADLs) were measured (with an informant) using the Functional Activities Questionnaire (FAQ) [79]. In addition to the neuropsychological battery, general cognitive status was measured using the Montreal Cognitive Assessment (MoCA) [80] and the Mini-Mental State Examination (MMSE) [81].

**Table 2:** Neuropsychological domains and tests by cohort.

**Aging cohort of PWH**				**AD spectrum cohort**			
***Domain***	***Assessment***	***HAND (n=23)***	***Controls (n=14)***	***Domain***	***Assessment***	***ADS (n=22)***	***Controls (n=16)***
**Learning**	**HVLT-R Learning Trials 1-3^1^**	**-1.60 (1.01)**	**0.24 (0.72)**	**Learning**	**HVLT-R Learning Trials 1-3^1^**	**-1.71 (1.39)**	**-1.13 (1.65)**
**Memory**	**HVLT-R Delayed Recall^1^**	**-1.61 (0.97)**	**0.25 (0.88)**		WMS-IV Logical Memory I Recall^1^	-1.23 (1.49)	-0.54 (1.63)
	**HVLT-R Recognition Discriminability Index^1^**	**-0.90 (1.12)**	**0.50 (0.58)**	**Memory**	**HVLT-R Delayed Recall^1^**	**-2.44 (1.33)**	**-1.72 (1.55)**
**Executive Function**	**Phonemic verbal fluency^1^**	**-0.53 (0.93)**	**0.14 (1.21)**		**HVLT-R Recognition Discriminability Index^1^**	**-2.44 (1.23)**	**-1.24 (1.68)**
	**Semantic verbal fluency^1^**	**-0.17 (1.02)**	**0.44 (1.09)**		WMS-IV Logical Memory II Delayed Recall^1^	-1.85 (1.43)	-1.02 (1.76)
	**Trail Making Test Part B^1^**	**-0.17 (0.74)**	**0.61 (0.81)**		WMS-IV Logical Memory II Recognition^1^	-0.54 (1.00)	-0.36 (1.04)
	Comalli Stroop Test Interference Trial^1^	-1.36 (1.63)	0.02 (1.30)	**Attention and Executive Function**	WAIS-IV Digit Span Forward, Backward, and Sequencing^1^	-0.74 (1.26)	-0.28 (1.21)
**Attention**	WAIS-III Symbol Search^1^	-0.36 (0.77)	1.33 (0.73)		**Trail Making Test Part B^1^**	**-0.88 (1.52)**	**-0.40 (1.31)**
	Comalli Stroop Test Word Trial^1^	-1.24 (1.29)	0.13 (0.84)	**Language**	Boston Naming Test^1^	-0.32 (1.34)	0.01 (1.31)
**Processing Speed**	WAIS-III Digit Symbol Coding^1^	-0.25 (0.70)	1.67 (0.74)		**Phonemic verbal fluency^1^**	**-0.70 (1.04)**	**-0.75 (1.22)**
	**Trail Making Test Part A^1^**	**-0.23 (0.83)**	**0.46 (1.00)**		**Semantic verbal fluency^1^**	**-1.66 (1.59)**	**-1.09 (2.02)**
	Comalli Stroop Test Color Trial^1^	-0.87 (1.05)	0.13 (0.84)	**Processing Speed**	WAIS-IV Digit Symbol Coding^1^	-0.50 (1.57)	0.16 (1.37)
**Motor**	Grooved Pegboard – Dominant Hand^1^	-1.18 (1.09)	0.20 (0.95)		**Trail Making Test Part A^1^**	**-1.21 (1.59)**	**-0.64 (1.57)**
	Grooved Pegboard – Non-Dominant Hand^1^	-1.27 (0.91)	0.16 (0.70)	**Functional Impairment**	Functional Activities Questionnaire (FAQ)^2^	12.23 (5.79)	0.19 (0.40)
**Functional Impairment**	Modified version of the Lawton and Brody Instrumental Activities of Daily Living Scale Total Declines^2^	1.65 (1.97)	1.00 (1.84)	**General Cognitive Status**	Montreal Cognitive Assessment (MoCA)^2^	20.86 (4.02)	22.78 (4.41)
					Mini-mental State Examination (MMSE)^2^	24.95 (3.34)	29.50 (0.73)
**Premorbid Function**	**WRAT-4 Word Reading^1^**	**-0.64 (0.86)**	**0.72 (1.05)**	**Premorbid Function**	**WRAT-4 Word Reading^1^**	**0.35 (0.91)**	**1.11 (0.80)**

Mean demographically corrected z-scores and standard deviations of neuropsychological performance on the assessments included in each study battery by group. Assessments that are shared across both cohorts are in bold. PWH, People with HIV; ADS, Alzheimer’s disease spectrum; HVLT-R, Hopkins Verbal Learning Test – Revised; WMS-IV, Wechsler Memory Scale, 4^th^ Edition; WAIS-III, Wechsler Adult Intelligence Scale, 3^rd^ Edition; WAIS-IV, Wechsler Adult Intelligence Scale, 4^th^ Edition; WRAT-4, Wide Range Achievement Test.

^1^z-score.

^2^Raw score.

### Florbetapir ^18^F positron emission tomography

Combined PET/CT data using ^18^F-florbetapir (Amyvid™, Eli Lilly) and a GE Discovery MI digital scanner (Waukesha, WI) were collected following the standard procedures described by the Society of Nuclear Medicine and Molecular Imaging (3D acquisition; single intravenous slow-bolus < 10 mL; dose = 370 MBq; waiting period = 30-50 min; acquisition = 10 min) [82]. Images were attenuation corrected using the CT data, reconstructed in MIMNeuro (slice thickness = 2 mm) [83], converted to voxel standardized uptake values based on body weight (SUVbw), and normalized into MNI space. Each scan was over-read by a fellowship-trained neuroradiologist blinded to group assignment and assessed as being “amyloid-positive” or “amyloid-negative” using established clinical criteria [83]. At this stage, patients who were amyloid-negative were excluded from the AD spectrum group.

### MEG experimental paradigm and behavioral analysis

During MEG recording, participants were seated in a nonmagnetic chair within a magnetically-shielded room and were monitored by study staff using a real-time audio-video feed from inside of the shielded room. Participants’ hands rested on a shelf attached to the chair and their right hand was positioned over a response glove that included a button for each of the second through fifth digits, although participants only used the buttons for the second and third digit (i.e., index and middle finger). Each participant performed 200 total trials of an arrow-based Eriksen flanker task (Figure 1) [36, 38]. Each trial began with a fixation cross that was presented for a variable duration of 1,450 to 1,550 milliseconds (ms), followed by a row of 5 arrows for 2,500 ms. Participants indicated whether the middle arrow was pointing to the left (right index finger) or right (right middle finger) as soon as possible following the onset of the target. Trials were pseudorandomized and equally divided between congruent and incongruent conditions, with left and right arrows being equally represented in each of the conditions. Trials with a reaction time 2.5 standard deviations (SDs) above or below each participant’s mean were excluded prior to averaging. Two by two ANCOVAs, controlling for the effect of age, were used to probe for group and condition differences in reaction time and accuracy. We first compared the behavioral metrics in healthy controls to those of patients (HAND + ADS) to identify commonalities across the diseases and then followed-up with ADS versus HAND comparisons to isolate disease specific deficits.

### MEG data acquisition

MEG data acquisition, structural coregistration, preprocessing, and sensor-/source-level analyses followed a pipeline similar to a number of previous manuscripts from our laboratory [84–86]. All recordings took place in a one-layer magnetically-shielded room with active shielding engaged for environmental noise compensation. A 306-sensor Elekta/MEGIN MEG system (Helsinki, Finland), equipped with 204 planar gradiometers and 102 magnetometers, was used to sample neuromagnetic responses continuously at 1 kHz with an acquisition bandwidth of 0.1–330 Hz. Each MEG dataset was individually corrected for head motion and subjected to noise reduction using the signal space separation method with a temporal extension (MaxFilter v2.2; correlation limit: 0.950; correlation window duration: 6 s) [87]. Only the gradiometer data was used in further analyses.

### Structural MRI processing and MEG coregistration

Prior to MEG acquisition, four coils were attached to the participants’ heads and localized, together with the three fiducial points and scalp surface, using a 3-D digitizer (Fastrak 3SF0002, Polhemus Navigator Sciences, Colchester, VT, USA). Once positioned in the MEG, the coils produced an electrical current with a unique frequency label and an accompanying measurable magnetic field, which allowed each coil to be localized in reference to the MEG sensor array throughout the recording. Since coil locations were also known in head coordinates, all MEG measurements could be transformed into a common coordinate system. With this coordinate system, each participant’s MEG data were co-registered with structural T1-weighted MRI data using BESA MRI (Version 2.0) prior to source-space analysis. Structural MRI data were aligned parallel to the anterior and posterior commissures and transformed into standardized space. Following source analysis (i.e., beamforming), each participant’s 4.0 × 4.0 × 4.0 mm functional images were also transformed into standardized space using the transform that was previously applied to the structural MRI volume and spatially resampled.

### MEG preprocessing, time–frequency transformation and sensor-level statistics

Cardiac and blink artifacts were identified in the raw MEG data and removed with signal-space projection (SSP), which was subsequently accounted for during source reconstruction [88]. The continuous magnetic time series was then bandpass filtered between 0.5 and 200 Hz, plus a 60 Hz notch filter, and divided into 2000 ms epochs, with the baseline extending from -450 to -50 ms prior to the onset of the stimulus. Epochs containing artifacts were rejected per participant using a fixed 3 MAD cutoff threshold. Briefly, in MEG, the raw signal amplitude is strongly affected by the distance between the brain and the MEG sensors, as the magnetic field strength falls off sharply as the distance from the current source increases. To account for this source of variance across participants, as well as other sources of variance, we used a 3 MAD threshold based on the within-subject signal distribution for both amplitude and gradient to reject artifacts. Across all participants, the average amplitude threshold for rejecting artifacts was 1042.51 (SD = 331.89) fT/cm and the average gradient threshold was 207.83 (SD = 122.86) fT/(cm*ms). Across all groups, an average of 165.77 (SD = 16.39) out of 200 possible trials per participant were used for further analysis in this experiment, including an average of 83.23 (SD = 8.53) out of 100 trials per participant in the incongruent condition and 82.54 (SD = 8.65) out of 100 trials per participant in the congruent condition. Importantly, our comparisons between groups and conditions were not affected by differences in the number of accepted trials per group, as this metric did not significantly differ as a function of condition (*p* = .255) or group (*p* = .142).

Complex demodulation [89, 90] was used to transform the artifact-free epochs into the time-frequency domain and the resulting spectral power estimations were averaged per sensor to generate time-frequency plots of mean spectral density. The time-frequency analysis was performed with a frequency-step of 1 Hz and a time-step of 50 ms between 4 and 50 Hz. These sensor-level data were then normalized by each respective bin’s baseline power for visualization purposes, calculated as the mean power during the -450 to -50 ms baseline period.

The specific time-frequency windows used for source imaging were determined by statistical analysis of the sensor-level spectrograms across all participants and both conditions using the entire array of gradiometers. Each data point in each sensor-level spectrogram was initially evaluated using a mass univariate approach based on the general linear model. To reduce the risk of false positive results while maintaining reasonable sensitivity, a two-stage procedure was followed to control for Type 1 error. In the first stage, paired sample *t*-tests against baseline were conducted on each data point and the output spectrogram of *t*-values was thresholded at *p* < .05 to define time-frequency bins containing potentially significant oscillatory deviations across all participants. In stage two, the time-frequency bins that survived the threshold were clustered with temporally and/or spectrally neighboring bins (per sensor) that were also above the threshold (*p* < .05), and a cluster value was derived by summing all of the *t*-values of all data points in the cluster. Nonparametric permutation testing was then used to derive a distribution of cluster values and the significance level of the observed clusters (from stage one) were tested directly using this distribution [91, 92]. For each comparison, 10,000 permutations were computed to build a distribution of cluster values. Based on these analyses, time-frequency windows within significant clusters (*p* < .001) were identified and used to guide source-level analysis. Cluster-based permutation testing was performed in BESA Statistics (v2.1).

### MEG source imaging

Cortical sources were imaged through an extension of the linearly constrained minimum variance vector beamformer [93–95], which employs spatial filters in the frequency domain to calculate source power for the entire brain volume. The single images were derived from the cross spectral densities of all combinations of MEG gradiometers averaged over the time-frequency range of interest, and the solution of the forward problem for each location on a grid specified by input voxel space. In principle, the beamformer operator generates a spatial filter for each grid point that passes signals without attenuation from the given neural region, while suppressing activity in all other brain areas. The filter properties arise from the forward solution (lead field) for each location on a volumetric grid specified by input voxel space, and from the MEG covariance matrix. For each voxel, a set of beamformer weights is determined, which amounts to each MEG sensor being allocated a sensitivity weighting for activity in the particular voxel. This set of beamformer weights is the spatial filter unique to the given voxel and this procedure is iterated until such a filter is computed for each voxel in the brain. Activity in each voxel is then determined independently and sequentially to produce a volumetric map of electrical activity with relatively high spatial resolution. In short, this method outputs a power value for each voxel in the brain, determined by a weighted combination of sensor-level time–frequency activity. Following convention, the source power in these images was normalized per participant using a prestimulus noise period (i.e., baseline) of equal duration and bandwidth [94]. MEG preprocessing and imaging used the Brain Electrical Source Analysis (version 7.0) software.

### Statistical analysis

3D maps of brain activity were averaged across all participants to qualitatively assess the anatomical basis of the significant oscillatory responses identified through the sensor-level analysis. Whole-brain flanker interference maps were then computed by subtracting the congruent condition image from the incongruent condition image for each time-frequency response per participant. Using the resulting interference maps, we employed an ANCOVA approach with age as a nuisance covariate. Our goals were to identify both similarities and differences in aberrant regional neural oscillatory activity during selective attention processing between the two clinical groups. Our first level analyses collapsed across patient groups (ADS + HAND) and aimed to identify regions where neural oscillatory responses differed between patients and controls. Brain regions where significant group differences were found were then probed to identify disease specific effects (i.e., ADS versus HAND). Bayes Factors (BF_01_) were also computed to evaluate the probability of the null for non-significant group comparisons between ADS and HAND. Our second level analyses were more exploratory and compared the ADS and HAND groups directly at the whole-brain level for each oscillatory response. All statistical maps used an initial uncorrected significance threshold of p < .001 with a volume threshold of 320 mm^3^.
